# Fed-Batch *mcl*- Polyhydroxyalkanoates Production in *Pseudomonas putida* KT2440 and Δ*phaZ* Mutant on Biodiesel-Derived Crude Glycerol

**DOI:** 10.3389/fbioe.2021.642023

**Published:** 2021-03-16

**Authors:** José Manuel Borrero-de Acuña, Manfred Rohde, Cesar Saldias, Ignacio Poblete-Castro

**Affiliations:** ^1^Institute for Microbiology, Technische Universität Braunschweig, Braunschweig, Germany; ^2^Braunschweig Integrated Centre of Systems Biology (BRICS), Technische Universität Braunschweig, Braunschweig, Germany; ^3^Central Facility of Microscopy, Helmholtz Centre for Infection Research, Braunschweig, Germany; ^4^Departamento de Química Física, Facultad de Química y Farmacia, Pontificia Universidad Católica de Chile, Macul, Chile; ^5^Biosystems Engineering Laboratory, Faculty of Life Sciences, Center for Bioinformatics and Integrative Biology, Universidad Andres Bello, Santiago, Chile

**Keywords:** *mcl*-poly(3-hydroxyalkanoates), fed-batch, crude glycerol, *pseudomonas putida*, PHA depolymerase

## Abstract

Crude glycerol has emerged as a suitable feedstock for the biotechnological production of various industrial chemicals given its high surplus catalyzed by the biodiesel industry. *Pseudomonas* bacteria metabolize the polyol into several biopolymers, including alginate and medium-chain-length poly(3-hydroxyalkanoates) (*mcl-*PHAs). Although *P. putida* is a suited platform to derive these polyoxoesters from crude glycerol, the attained concentrations in batch and fed-batch cultures are still low. In this study, we employed *P. putida* KT2440 and the hyper-PHA producer Δ*phaZ* mutant in two different fed-batch modes to synthesize *mcl*-PHAs from raw glycerol. Initially, the cells grew in a batch phase (μ_*max*_ 0.21 h^–1^) for 22 h followed by a carbon-limiting exponential feeding, where the specific growth rate was set at 0.1 (h^–1^), resulting in a cell dry weight (CDW) of nearly 50 (g L^–1^) at 40 h cultivation. During the PHA production stage, we supplied the substrate at a constant rate of 50 (g h^–1^), where the KT2440 and the Δ*phaZ* produced 9.7 and 12.7 gPHA L^–1^, respectively, after 60 h cultivation. We next evaluated the PHA production ability of the *P. putida* strains using a DO-stat approach under nitrogen depletion. Citric acid was the main by-product secreted by the cells, accumulating in the culture broth up to 48 (g L^–1^) under nitrogen limitation. The mutant Δ*phaZ* amassed 38.9% of the CDW as *mcl*-PHA and exhibited a specific PHA volumetric productivity of 0.34 (g L^–1^ h^–1^), 48% higher than the parental KT2440 under the same growth conditions. The biosynthesized *mcl-*PHAs had average molecular weights ranging from 460 to 505 KDa and a polydispersity index (PDI) of 2.4–2.6. Here, we demonstrated that the DO-stat feeding approach in high cell density cultures enables the high yield production of *mcl*-PHA in *P. putida* strains using the industrial crude glycerol, where the fed-batch process selection is essential to exploit the superior biopolymer production hallmarks of engineered bacterial strains.

## Introduction

The worldwide manufacture of petrochemical plastics reaches over 359 million tons annually as these materials are essential in our current lifestyle (Poblete-Castro et al., 2020a; Tournier et al., 2020). Unfortunately, synthetic plastics are not prone to biodegradation and massively accumulate in the oceans and soil ecosystems (Zettler et al., 2013; Lau et al., 2020). Factors like temperature and radiation decompose the polymers in small particles (<5 mm), entering now into the food chain (Law and Thompson, 2014). This phenomenon is detrimental to preserving natural environments and human health (Smith et al., 2018), demanding actions to move toward a circular bioproduction economy. To this end, commercial substitutes of petrochemical plastics are the biodegradable poly(3-hydroxyalkanoates) (PHAs). These biopolymers possess physical and mechanical properties similar to oil-based plastics displaying thermal malleability and elasticity and having suitable breaking points for thermoforming (Laycock et al., 2013). The industrial sectors that exploit these biopolymers include food, textile, agriculture, biomedicine, and electronics (Raza et al., 2018).

PHAs are naturally occurring inclusion bodies during nutrient imbalance formed in microbes’ cytosolic space and reported as energy reservoirs (Madison and Huisman, 1999) and essential elements to cope with different stress agents (Obruca et al., 2017). A manifold of companies currently manufactures PHAs at a large scale (Borrero-de Acuña et al., 2017). However, industrial production of PHAs is costly, given high operational expenses because of the employed carbon substrate and downstream processing (Chen et al., 2020). Hence, to overcome these setbacks, waste materials arise as low-cost carbon substrates to sustain bacterial growth and renewable polyester production (Cesário et al., 2014; Nielsen et al., 2017; Borrero-de Acuña et al., 2019). Combining engineered microbial cell factories with high cell density fermentations is a robust approach to achieve elevated PHA productivities (Choi et al., 2020).

In the last decade, the biodiesel industry has generated large quantities of crude glycerol, an inevitable by-product resulting from the esterification process of fatty acids (Garlapati et al., 2016). Given the high glycerol surplus, the product market price is continually dropping (Zhang et al., 2020), making it an attractive substrate to derive biochemicals (Kaur et al., 2020). Microbial catabolism of crude glycerol presents some challenges since the biodiesel by-product contains methanol, traces of diesel, and heavy metals (Mothes et al., 2007; Samul et al., 2014). Remarkably, *Pseudomonas* strains can endure these toxic materials (Poblete-Castro et al., 2017) and fuel glycerol metabolic products into PHA biosynthetic pathways enabling the synthesis of *medium-chain* length (*mcl*-PHAs) (Kenny et al., 2012; Pappalardo et al., 2014; Fu et al., 2015; Liu et al., 2018) and copolymers of short-*co*-medium-chain length polyesters (Orellana-Saez et al., 2019; Pacheco et al., 2019). Batch and fed-batch production of *mcl*-PHA is feasible using crude glycerol in *Pseudomonas putida* where the strain KT2440 formed 34% of the cell dry weight (CDW) as polyester with a final product titer of 1.45 (g L^–1^) in 75 h (Poblete-Castro et al., 2014a). A newly isolated soil strain from Thailand, *Pseudomonas* sp. ASC2, proved to synthesize 3.02 (g L^–1^) of *mcl*-PHAs in flask experiments. In a high cell density fermentation, *P. putida* GO16 attained 19 (g L^–1^) biomass using the waste polyol, showing a specific PHA volumetric productivity of 0.13 (gPHA L^–1^ h^–1^) (Kenny et al., 2012).

While the polymerization of PHAs in *P. putida* is well characterized and relies on the PHA synthase proteins PhaC1 and PhaC2, the not yet fully understood depolymerization process is a result in part of the enzymatic action of PHA depolymerase (PhaZ, PP_5004) (Arias et al., 2013). Different metabolic stimuli govern gene regulatory crosstalk, with the polymerization vs. depolymerization of PHA kinetics still unclear (Karmann et al., 2017; Velázquez-Sánchez et al., 2020). The accumulating evidence suggests that the polymerization regulatory control depends on the type of carbon substrate (De Eugenio et al., 2010). Inactivation of the *phaZ* gene in *P. putida* KT2442 boosted *mcl*-PHA production of cells grown on fatty acids (Cai et al., 2009). Conversely, non-related PHA carbon sources, glucose or gluconate, did not yield higher PHA titers in this mutant. Glycerol-grown cells of a *phaZ*-lacking strain of KT2440 produced 36% more biopolymer than the wild type using crude glycerol (Poblete-Castro et al., 2014a). Using fatty acids as substrates, a *phaZ* minus strain of KT2440 formed more than 70 (g L^–1^) of unsaturated *mcl*-PHA in fed-batch cultures (Vo et al., 2015). These experiments laid the groundwork for challenging the production capacities of the *phaZ*-deficient mutant in high cell density cultures. Here, we assessed different feeding strategies for high titer PHA production in *P. putida* strains on industrial crude glycerol. The DO-stat fed-batch fermentation is best suited to bioconvert raw glycerol into the elastomer polyesters where the Δ*phaZ* mutant and the parental KT2440 reached *mcl*-PHA specific volumetric productivities of 0.34 and 0.23 (g L^–1^ h^–1^), respectively.

## Results and Discussion

### Constant-Feeding PHA Synthesis Under Nitrogen Limitation

**FIGURE 1:**
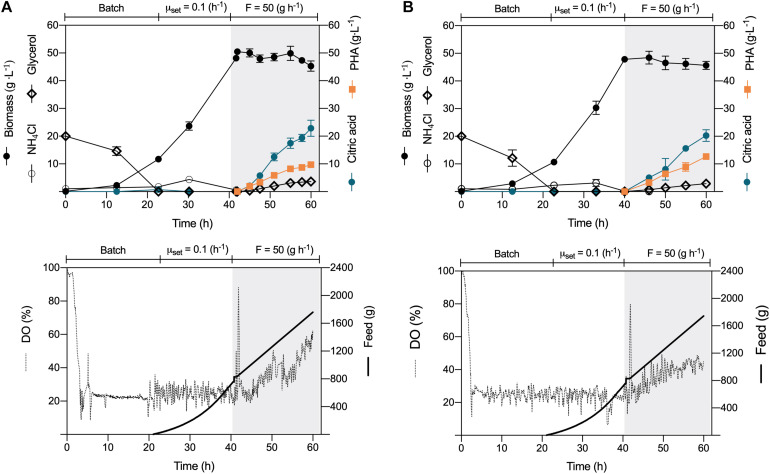
Constant-feeding fed-batch *mcl*-PHA production. The fermentation process comprised three phases: (i) batch, (ii) exponential feeding, and (iii) constant feeding of crude glycerol (50 g h^–1^) under nitrogen depletion. Time profile of **(A)** *P. putida* KT2440 and **(B)** *phaZ*-knockout mutant. The data represent the mean values and standard deviation from two independent experiments.

*P. putida* KT2440 and a *phaZ*-deficient mutant strain demonstrated in a previous study to synthesize efficiently *mcl*-PHAs from crude glycerol in batch cultures (Poblete-Castro et al., 2014a). We now challenged these natural polyester producers in the process of choice for the industrial production of PHAs, the fed-batch culture. Applying different feeding strategies using industrial crude glycerol without any modification (Cremer Oleo, GmbH, Germany), we developed a three-stage fermentation where the two initial phases aimed to form biomass and the third stage *mcl*-PHA under nitrogen depletion. The batch cultures started with a biomass concentration of 0.13 (g L^–1^) in a 4 L working volume bioreactor. The culture broth initially contained 20 (g L^–1^) crude glycerol and 1 (g L^–1^) of glucose to prevent a characteristic extended lag phase (Escapa et al., 2012; Beckers et al., 2016), usually taking more than 10 h of *P. putida* cells growing on glycerol as the sole C source (Poblete-Castro et al., 2020b). As the *P. putida* cells propagate, foam developed in the bioreactor (at 3 h), which dispersed once we provided antifoam (200 μL L^–1^). After 22-h cultivation, *P. putida* KT2440 and Δ*phaZ* mutant had similar maximum specific growth rates of 0.21 h^–1^ (Figure 1A), reaching a CDW of 11.5 g L^–1^ (Figures 1A,B). Then, we started feeding glycerol exponentially, setting the specific growth rate at nearly 50% of μ_*max*_ (0.1 h^–1^) (Figure 1). During this phase, no glycerol accumulated in the culture broth or the cells secreted by-products due to the coupled catabolism and anabolism under carbon limiting conditions (Poblete-Castro et al., 2012; González-Cabaleiro et al., 2015).

At 40 h cultivation, we no longer provided glycerol and ammonium; we instead provided a substrate pulse to attain 10 (g L^–1^) of glycerol within the bioreactor. This procedure triggered nitrogen limitation, and once glycerol was under the detection limit (after 1 h), we fed the substrate, but this time at a constant mass flow rate of 50 (g h^–1^). During the biopolymer production phase, we upheld the airflow rate, providing only filter air instead. The DO evolution showed a marked increase as the cells accumulated higher amounts of PHAs through the process, showing a reduced O_2_ demand as the cells no longer duplicate (Figures 1A,B). The *P. putida* strains secreted citric acid as the main co-product as glycerol accumulated in the medium, showing a final yield on the polyol of 0.18 ± 0.01 (g g^–1^) and 0.16 ± 0.02, for the wild-type and the mutant strain, respectively (Table 1 and Figure 1). Indeed, nitrogen depletion of glycerol-grown *P. putida* cells inhibits the TCA cycle enzymes like isocitrate dehydrogenase (ICD), slowing the carbon flux through the oxidative route (Beckers et al., 2016). The biomass suffered a slight reduction in KT2440 and the *phaZ*-knockout mutant from ∼50 (g L^–1^) to 45.4 and 46.7 (g L^–1^), respectively, a common trend of cells enduring overflow metabolism (Xu et al., 1999; Poblete-Castro et al., 2014b). At the end of the fermentation process (60 h), the wild-type KT2440 synthesized 9.7 (gPHA L^–1^) with a biopolymer content of 21.4% of the CDW (Figure 1A and Table 1), while the Δ*phaZ* deletion mutant achieved 12.7 (gPHA L^–1^), amassing 27.2% of the cell biomass as polyester (Figure 1B and Table 1). Finally, the monomeric composition of the generated biopolymers by constant-feeding strategy was dominated by 3-hydroxydecanoate with a 75.4 and 76.1% relative molar fraction in the wild-type and Δ*phaZ* mutant, respectively (Table 1). The rest of the monomers had the following decreasing proportion within the polymeric chain in both tested strains: 3-hydroxyoctanoate, 3-hydroxydodecanoate, 3-hydroxy-5-cis-dodecanoate, 3-hydroxyhexanoate, and 3-hydroxytetradecanoate.

**TABLE 1:** Medium-chain length (*mcl*-PHA) production in *Pseudomonas* strains under various fermentation modes utilizing crude glycerol.

Strain	Production mode	Biomass	PHA	PHA content	Y_CIT/Gly_ Citrate yield	Y_PHA/Gly_ PHA yield	Specific PHA volumetric productivity	Monomeric composition (%)						References
	
		(g L^–1^)	(g L^–1^)	(%wt)	(g g^–1^)	(g g^–1^)	(g L^–1^ h^–1^)	C6	C8	C10	C12	C12:1	C14	
*P. putida* KT2440	Constant feeding	45.3	9.7	21.4	0.18	0.08	0.16	1.2	15.3	75.4	5.7	2.4	0.3	This study
Δ*phaZ*	Constant feeding	46.7	12.7	27.2	0.16	0.10	0.21	1.3	16.1	76.1	5.9	0.6	N.D.	This study
*P. putida* KT2440	DO-stat	49.5	13.8	27.9	0.36	0.11	0.23	0.8	15.8	73.3	5.6	4.5	0.4	This study
Δ*phaZ*	DO-stat	52.4	20.4	38.9	0.32	0.13	0.34	1.2	16.7	76.2	5.4	0.5	N.D.	This study
*P. putida* GO16	Fed batch	19.0	6.3	33.2	N.D.	N.S	0.13	3	18	35	13	15	7	Kenny et al., 2012
*Pseudomonas* sp. ASC2	Batch	10.7	3.0	28.2	N.D	N.S.	0.04	N.D.	4.8	N.D.	N.D.	95.2	N.D.	Muangwong et al., 2016

*N.D., Not detected; N.S., Not shown.*

### PHA Production Using a Dissolved-Oxygen-Stat Feeding (DO-Stat) Under Nitrogen Limitation

**FIGURE 2:**
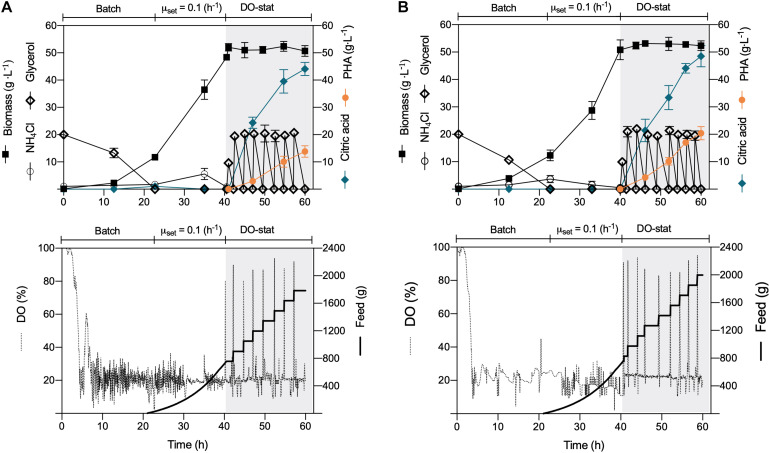
DO-stat fed-batch *mcl*-PHA production. The culture comprised three phases: (i) batch, (ii) exponential feeding, and (iii) DO-stat response for substrate feeding under nitrogen limitation. Time profile of **(A)** *P. putida* KT2440 and **(B)** *phaZ*-knockout mutant. The data represent the mean values and standard deviation from two independent experiments.

Despite the sound PHA volumetric concentration obtained in this study operating the constant-feeding scheme, the *P. putida* strains did not amass the PHA content previously reported in batch cultures (Poblete-Castro et al., 2014a). Thus, to fully harness the biopolymer production capacities of the Δ*phaZ* mutant and the wild type, a dissolved oxygen-stat (DO-stat) feeding approach was applied. This well-controlled strategy has proven to yield higher PHA amount and content in engineered *P. putida* strains on glucose and aromatics (Poblete-Castro et al., 2014b; Borrero-de Acuña et al., 2020). For biomass formation, we repeated the same growth conditions as those employed during the constant-feeding approach, where a mixture of pure oxygen and air was provided to avoid oxygen limitation. Nitrogen was always sufficient at this stage, reaching both strains biomass productions of nearly 50 (g L^–1^) (Figure 2). At this point, we provided a pulse of glycerol (10 g L^–1^) to enable the cells to consume the remaining ammonium, and to get a better DO response, we provided only filter air as carried out in the constant-feeding cultivations. Likewise, citrate began to accumulate in the culture broth due to the metabolic shift provoked by nitrogen limitation (Figure 2). As a response to glycerol exhaustion, the DO saturation increased; thus, every time the dissolved oxygen exceeded a value of 70%, an automated addition of the substrate (20 g L^–1^ glycerol) occurred in an interval of 15 min (Figure 2). This feeding operation did not reduce the formed biomass, and the intracellular polyester synthesis boosted, maintaining a rapid pace of accumulation until the termination of the process (Figure 2). After 20 h of feeding driven by the DO response, the wild-type strain attained a biomass titer of 49.5 (gCDW L^–1^), of which 13.8 (g L^–1^) consisted of *mcl*-PHA (Figure 2A and Table 1). Similarly, the depolymerase-deficient mutant achieved a biomass yield of 52.4 (gCDW L^–1^) but comprised an enhanced biopolymer yield of 20.4 (g L^–1^)—overall PHA content 38.9%wt (Figure 2B). The specific PHA volumetric productivity (0.34 g L^–1^ h^–1^) reached by the Δ*phaZ* mutant is the highest reported today to derive *mcl*-PHA using industrial crude glycerol (Table 1). *P. putida* KT2440 and its *phaZ* knockout mutant strain secreted 48 (g L^–1^) of citric acid in the DO-stat stage (Figure 2). However, citrate yields on glycerol obtained in the fed-batch cultivations were lower (Table 1) than the values previously reported (0.5 g g^–1^) in batch cultures (Poblete-Castro et al., 2014a). The co-production of citrate not only is detrimental in terms of carbon loss for biopolymer synthesis but also impacts negatively to the PHA production process as production costs boost, and the addition of the base solution to maintain the optimal pH exerts a dilution effect lowering the PHA volumetric productivities (Warnecke and Gill, 2005).

It is clear that further metabolic engineering efforts must avoid the entry of the carbon flux into the TCA cycle during the biopolymer production phase to diminish organic acid formation in *P. putida* KT2440. There are several routes to achieve this, beginning with the overexpression of acetyl-CoA carboxylase (ACC), which converts acetyl-CoA into malonyl-CoA, where the latter is the main precursor for PHA synthesis of substrates metabolized through the central carbon metabolism (Rehm et al., 1998; Poblete-Castro et al., 2013). Another path is to block the entry of acetyl-CoA into the Krebs cycle by inactivating *in vivo* the citrate synthase enzyme using RNA interference or antisense RNA (Desai and Papoutsakis, 1999; Ko et al., 2020), as the genetic systems can be activated during the nitrogen-limiting phase, which may have no negative impacts on biomass formation in the initial nutrient-sufficient phase. Finally, inspection of the monomer composition of the biosynthesized PHA highly resembled the previous feeding regime’s values. The most abundant hydroxy acid encountered in both strains was the C10 (Table 1). This monomer’s relative molar fraction in the parental strain was 73.3%, whereas the Δ*phaZ* knockout mutant presented 76.2% (Table 1).

### Visualization of the Biosynthesized *mcl*-PHA and Physical Properties

**FIGURE 3:**
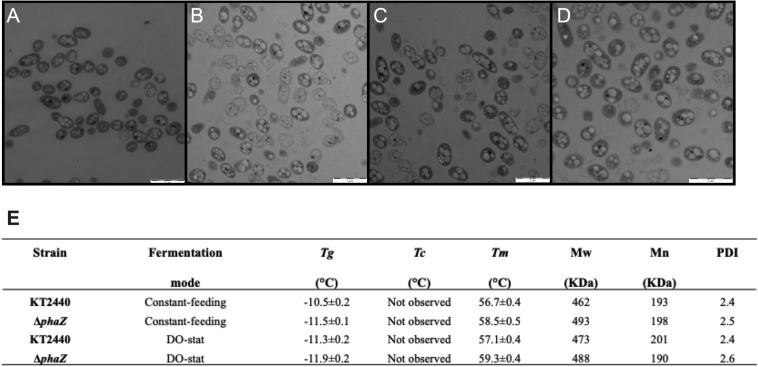
Transmission electron micrographs of *P. putida* cells in fed-batch fermentation taken at 60 h fermentation. **(A)** *P. putida* KT2440 constant feeding, **(B)** *phaZ*-knockout mutant constant feeding, **(C)** *P. putida* KT2440 DO-stat, and **(D)** *phaZ*-knockout mutant DO-stat. **(E)** Thermal properties of the biosynthesized *mcl*-PHA: Transition glass temperature (*Tg*), melting temperature (*Tm*), and crystallization temperature (*Tc*). Molecular weight (*Mw*), average-number molecular weight (*Mn*), and polydispersity index (PDI). The values represent mean and standard deviation from two replicates.

Crude glycerol contains harmful compounds, including methanol, salts, and heavy metals (Mothes et al., 2007; Samul et al., 2014). These elements impair cell growth and evoke stress responses at the proteome and transcriptome levels (Manara et al., 2012; Fu et al., 2015; Bojanovič et al., 2017). This toxic feedstock might also influence cell morphology and the polymerization process of the polyesters. Figure 3 depicts micrographs acquired by transmission electron microscopy taken at the maximum PHA formation point (60 h) in the constant feeding (3A, KT2440 and 3B, Δ*phaZ mutant*) and DO-stat fermentations (3C, KT2440 and 3D, Δ*phaZ mutant*). Neither the parental strain nor the *phaZ*-disrupted mutant displays significant cellular morphological variance or altered PHA inclusion bodies (Figure 3). Conversely to a previous study on crude glycerol, the cells showed no aggregation (Poblete-Castro et al., 2014a), indicating robust growth and proper mixing. Impurities present in the substrate source as black dots were distinctive in both growing *P. putida* strains. Notably, intracellular PHA structures had no apparent alteration by deleting the polymer disassembling enzyme PhaZ (Figures 3B,D). As previously noted, *P. putida* strains grown on crude glycerol presented larger numbers of intracellular PHA granules than those cultivated on pure glycerol (Poblete-Castro et al., 2014a), which are more unevenly shaped (Figures 3A–D). The PHA morphology depends on various factors, including the level of expression of granular associate proteins like phasins and PHA polymerase and depolymerase (Jendrossek, 2009), which in turn rely on the culture conditions and the growth substrate (De Eugenio et al., 2010). Under stress, PHA-producing bacteria alter the PHA content and its morphology as a response mechanism improving the survival rate when cells thrive under high salt concentrations and low and elevated temperatures or encountering toxic compounds (Obruca et al., 2020). As raw glycerol contains methanol and heavy metal, this could explain the intracellular granule’s observed alterations.

We also unveiled the thermal properties of the biosynthesized *mcl*-PHA via differential scanning calorimetry (DSC) of purified biopolymer sampled at 60-h cultivation from the bioreactors. The obtained glass transition points (*Tg*) were very similar among the produced *mcl*-PHAs (−10.5 to −11.9°C) showing a single *Tg* characteristic of a copolymer of medium-chain length polyesters (Cheema et al., 2012; Pacheco et al., 2019; Figure 3E). As distinctive elastomers, the melting endotherms were in the range of 56.7–59.3°C (Chan Sin et al., 2010; Gumel et al., 2012). Most importantly, the molecular weight of the purified PHA was higher than 400 KDa (Figure 3E), a prerequisite for processing these kinds of macromolecules industrially (Mothes et al., 2007), especially as softener materials (Muangwong et al., 2016; Liu et al., 2018). Together, the PHA thermal and physical properties did not vary significantly between the polyesters synthesized by wild-type and the *phaZ* minus mutant (Figure 3E). A *Pseudomonas resinovorans* strain lacking the *phaZ* gene presented the same molecular weights and thermal properties of the accumulated PHA as the parental strain (Solaiman et al., 2003). Further, given the high polydispersity index values (PDI > 2), the synthesized *mcl*-PHA on crude glycerol would also perform appropriately in extruders as the elastomers exhibit narrow average-number molar mass distribution (Mn, 190 KDa), where these values are relatively close to commercial polyesters (Debuissy et al., 2017). While extracting the PHA from the cells, we could appreciate the elastomer film that remained after chloroform evaporation in a Petri dish for purification. Film materials are paramount in the packaging and agroindustry sectors. A filmable *mcl*-PHA was synthesized by *Pseudomonas mediterranea* using raw glycerol (Pappalardo et al., 2014). The obtained *mcl*-PHA presented nearly the same proportion of C10 in the monomeric chain than the filmable biopolymer, and above all, both elastomers display *Tm* values higher than 50°C and Mw of 400 KDa.

## Conclusion

In this study, we demonstrated that the applied feeding strategy during *mcl*-PHA synthesis in *P. putida* strains using the crude glycerol as substrate influences the amount of formed polyester but not their physical properties. We proved that a DO-stat fed-batch process is a more suitable feeding strategy than the constant-feeding approach to metabolize the toxic crude glycerol resulting in nearly 50% more biopolymer at the end of the fermentation in the *phaZ*-deficient mutant and the wild-type KT2440. The attained specific PHA volumetric productivity by Δ*phaZ* knockout mutant (0.34 g L^–1^ h^–1^) is a step further in the quest to derive *mcl*-PHA from the polyol of the biodiesel industry in a more cost-effective fashion. There is still room for improvement concerning PHA production since the *P. putida* cells secreted high citric acid levels. Further metabolic engineering endeavors must reduce the carbon wastage and redirect carbon flux toward polyester biosynthetic pathways to mining the cell factory’s PHA production performance.

## Methods

### *P. putida* Strains

The wild-type *P. putida* KT2440 (DSM 6125) was obtained from the DSMZ collection, Germany, and the Δ*phaZ* mutant was constructed in a previous study (Poblete-Castro et al., 2014a). These strains were used for the different fed-batch fermentations.

### Culture Conditions

Strains were stored in 25% glycerol at −80°C as glycerol stocks. Cells were routinely streaked onto Luria–Bertani (LB) agar plates and grown overnight to isolate single colonies. For any shake flask cultivation, *P. putida* strains were aerobically grown at 180 rpm and 30°C. Single colonies were picked from the plate and transferred into a 50 mL shake flask containing 10 mL of LB liquid medium. Defined minimal medium (M9) containing 6 (g L^–1^) crude glycerol (Cremer Oleo, GmbH, Hamburg, Germany) was employed for subsequent pre-culture cultivation. The industrial glycerol contains 80% glycerol, 0.5% methanol, 10% ash, 3% organic matter, and 6.5% water. The M9 medium composition consisted (per liter) of 12.8 g Na_2_HPO_4_⋅7H_2_O, 3 g KH_2_PO_4_, 4.7 g (NH_4_)_2_SO_4_, and 0.5 g NaCl. After autoclave sterilization, the medium was supplemented with filtered trace elements [6.0 FeSO_4_⋅7H_2_O, 2.7 CaCO_3_, 2.0 ZnSO_4_⋅H_2_O, 1.16 MnSO_4_⋅H_2_O, 0.37 CoSO_4_⋅7H_2_O, 0.33 CuSO_4_⋅5H_2_O, and 0.08 H_3_BO_3_ (mg L^–1^)] and 0.12 g of MgSO_4_⋅7H_2_O. Ten milliliters of M9 medium in a 50 mL shake flask was inoculated with the overnight LB-grown culture at an initial OD at 600_*nm*_ of 0.2 and incubated overnight. A second pre-culture was initiated by transferring a predetermined volume of the previous one into 300 mL of M9 medium containing 10 g L^–1^ crude glycerol in 1 L baffled Erlenmeyer flasks and cultivated overnight.

### Fed-Batch Cultivations

The fed batches were seeded with the second pre-culture to attain an initial cell density of (OD_600_ 0.26). The fed-batch reactor contained M9 medium supplemented with 20 g L^–1^ crude glycerol, 0.12 g L^–1^ MgSO_4_⋅7H_2_O, and 8 ml of the trace element solution. Overall, 4 L of working volume was set up in a 15 L vessel (B10 stirred tank bioreactor, Biologische Verfahrenstechnik, Basel, Switzerland) to conduct the fermentation processes. The airflow rate was maintained at 10 L min^–1^ (air-to-pure oxygen ratio was set to 10:1) during the biomass production phase. In the PHA production phase, the airflow rate was kept, but pure oxygen was no longer needed, and the reactor was sparged only with compressed air, providing better DO response when the carbon substrate was supplied. The temperature was set at 30°C and 12.5 (w/v) of NH_4_OH was added as required to stabilize pH to 6.8 ± 0.1 during the course of the biomass formation phase and as nitrogen supply to avoid N limitation. Then, in the PHA production phase, the base was replaced by NaOH 10% (w/v). When required, Tego Antifoam (Evonik, Germany) was supplemented to prevent foaming (200 μL L^–1^). The agitation speed was automatically adjusted to 800 rpm in order to keep the dissolved oxygen level above 20% air saturation.

The feeding solution contained per liter: 780 g crude glycerol and 12 g MgSO_4_⋅7H_2_O. An exponential feeding strategy was applied during the biomass production phase, following an exponential function (Eq. 1).

(1) $$F\left(t\right)=\mu_{s\,e\,t}\left(V_{0}.X_{0}\right)e^{\left(\mu set.t\right)}{\left(S_{0}.Y_{x\,s}\right)}^{-1}$$

Hereby, *F* is the feed rate (L h^–1^), *μ*_*set*_ is the desired specific growth rate (set to 0.1 h^–1^), *S*_0_ is the substrate concentration of the feed medium (780 g L^–1^ crude glycerol), *t* is the time after feed start (h), *Y*_*xs*_ is the biomass yield on crude glycerol taken from Poblete-Castro et al. (2014a), *V*_0_ is the initial volume of the culture (L), and *X*_0_ is the initial biomass level (g cells L^–1^).

### Biomass Quantification and Analytical Procedures

The optical density (OD_600 nm_) was registered over time in a spectrophotometer (Ultraspec 2000; Hitachi, Japan) to determine cell growth. Ten milliliters of cells was harvested at 9,000×*g* for 10 min at 4°C and washed once with distilled water prior to CDW gravimetric quantification in pre-weighed tubes. The cell pellet was dried to constant weight at 100°C. A photometric test (LCK 303 kit, Hach Lange, Danaher, United States) served to measure offline the ammonium levels in the supernatant. Supernatant samples were withdrawn and accordingly diluted to analyze the crude glycerol and organic acid (citrate, isocitrate, succinate, fumarate, malate, pyruvate, and oxaloacetate) concentrations by HPLC Agilent 1260 (Agilent, Krefeld, Germany). The HPLC system was equipped with an 8-mm Rezex ROA-organic acid H column (Phenomenex, United States), which was operated at 65°C. The mobile phase consisted of 0.013 N H_2_SO_4_ at a 0.5 ml min^–1^ flow coupled with a RID detector system (Agilent serie1260).

### PHA Characterization and Quantification

The polyesters were firstly methanolized in order to determine the PHA monomeric compositions and the intracellular PHA content. For this, 10 ml of culture was transferred to a falcon tube and cells were harvested at 9,000 × *g* for 10 min at 4°C (Eppendorf 5810 R, Hamburg, Germany). Pellets were washed once with distilled water. The supernatants were discarded, and the pelleted cells were stored at −20°C until needed. The methanolysis procedure was performed as previously specified (Borrero-de Acuña et al., 2014). Gas chromatography (GC) coupled with mass spectrometry (MS) was used to analyze the methyl esters of monomers. One milliliter of the organic phase was injected into a Varian GCMS system 450GC/240MS ion trap mass spectrometer (Varian Inc., Agilent Technologies) at a split ratio of 1:10. The software employed to process the resulting data was the MS Workstation 6.9.3 (Varian Inc., Agilent Technologies). The different compounds, i.e., the methyl esters of 3-hydroxyexanoate, 3-hydroxyoctanoate, 3-hydroxydecanoate, 3-hydroxydodecanoate, 3-hydroxy-5-cis-dodecanoate, and 3-hydroxytetradecanoate, were split by using a FactorFour VF-5ms capillary column (30 m × 0.25 mm i.d. × 0.25 mm film thickness), including calibration with commercial PHB (Sigma−Aldrich, MI, United States) and purified *mcl*−PHA from a previous work (Oliva-Arancibia et al., 2017). The carrier gas helium was set to a flow rate of 0.9 ml min^–1^. The temperatures for the injector and transfer line were established at 275 and 300°C, respectively. The oven temperature was stepwise programmed as follows: 40°C for 2 min, rising progressively from 40 to 150°C at a rate of 5°C min^–1^ and ultimately increasing to 280°C at a constant rate of 10°C min^–1^. To capture positive ions, an electron ionization at 70 eV was settled, while the mass spectra were registered by scanning ions of m/z 50 to m/z 650. The PHA concentration was determined by the method described by Lageveen et al. (1988). The percentage of biopolymer in relation with the CDW in two biological replicates was averaged out to ascertain PHA content (wt%).

### Transmission Electron Microscopy

Prior to fixation, the bacteria were cooled down to 4°C. Next, 2% of glutaraldehyde and 5% of formaldehyde (5%) were added. Cells were subsequently washed with cacodylate buffer (0.01 mol L^–1^ cacodylate, 0.01 mol L^–1^ CaCl_2_, 0.01 mol L^–1^ MgCl_2_ 6H_2_O, and 0.09 mol L^–1^ sucrose, pH 6/9) and stained for 1 h at room temperature with 1% aqueous osmium solution. Dehydration was achieved by adding acetone at increasing concentrations (10, 30, 50, 70, 90, and 100%) and incubating the samples for 30 min at each time. Solely the dehydration with 70% acetone containing 2% uranyl acetate was allowed overnight. The Spurr formula for hard resin was applied to infiltrate samples with an epoxy resin. A diamond knife was used to slice the samples into ultra-thin sections, which were further counterstained with a mixture of uranyl acetate and lead citrate. A TEM910 transmission electron microscope (Carl Zeiss, Oberkochen, Germany) was operated at an acceleration voltage of 80 kV to acquire images. Digital imaging of ultra-thin sections was acquired with a Slow-Scan CCD-Camera (ProScan, 1,024 × 1,024, Scheuring, Germany) with ITEM-Software (Olympus Soft Imaging Solutions, Munster, Germany).

### Differential Scanning Calorimetry Analysis

The glass transition, crystallization, and melting temperatures (*Tg*, *Tc*, and *Tm*, respectively) of each sample were determined by a Mettler–Toledo DSC 821e. The following five cycles were performed: (i) a first heating from −40 to 200°C at 10°C min^–1^, (ii) an isotherm for 3 min, (iii) a cooling from 200 to −40°C at 10°C min^–1^, (iv) an isotherm for 3 min, and (v) a second heating from −40 to 200°C at 10°C min^–1^.

### Size Exclusion Chromatography Measurements

The weight average molecular weights (Mw) and the respective polydispersity indices of the samples were determined by size exclusion chromatography (SEC) along with a static light scattering Dawn EOS in line with an Optilab DSP interferometric refractometer (both were obtained from Wyatt Technology) using CHCl_3_ as the mobile phase and a calibration curve constructed using polystyrene standard samples. The SEC measurement was performed on a Dionex P590A liquid chromatography pump equipped with a guard column and two PLgel 5-mm Mixed C (300 × 7.5 mm) columns in series with a Viscotek differential refractometer. The eluent was CHCl_3_ at a flow rate of 1.0 mL min^–1^ at 25°C. Polystyrene standards with a molecular weight range of 1,020–1,944,000 were used to generate a universal calibration curve.

## Data Availability Statement

The original contributions presented in the study are included in the article/supplementary material, further inquiries can be directed to the corresponding author/s.

## Author Contributions

IP-C conceived and performed the fed-batch fermentation and analytics. CS performed the thermal analysis. MR carried out the TEM studies. JB-dA and IP-C interpreted the data and wrote the manuscript. All authors contributed to the article and approved the submitted version.

## Conflict of Interest

The authors declare that the research was conducted in the absence of any commercial or financial relationships that could be construed as a potential conflict of interest.
